# A Review on Bioactivities of Tobacco Cembranoid Diterpenes

**DOI:** 10.3390/biom9010030

**Published:** 2019-01-16

**Authors:** Ning Yan, Yongmei Du, Xinmin Liu, Hongbo Zhang, Yanhua Liu, Zhongfeng Zhang

**Affiliations:** Tobacco Research Institute of Chinese Academy of Agricultural Sciences, Qingdao 266101, China; duyongmei@caas.cn (Y.D.); liuxinmin@caas.cn (X.L.); zhanghongbo@caas.cn (H.Z.); liuyanhua@caas.cn (Y.L.)

**Keywords:** tobacco, cembranoid diterpenes, bioactivity, biocatalysis, semisynthesis

## Abstract

Cembranoids are carbocyclic diterpenes comprising four isoprene units and are natural products with a parent skeleton consisting of a 14-membered ring. They have gained wide interest in recent years and are a major hotspot in the research of natural product chemistry. Since 1962, various tobacco cembranoid diterpenes have been identified. This review systematically discusses and summarises the excellent antimicrobial, insecticidal, cytotoxic and neuroprotective activities of tobacco cembranoid diterpenes. These compounds show potential to be developed as botanical fungicides, cytotoxic drugs and drugs for the treatment of human immunodeficiency virus, Alzheimer’s disease, Parkinson’s disease, and other neurodegenerative diseases. However, there are relatively few studies on the structure–activity relationship (SAR) of tobacco cembranoid diterpenes. Therefore, future studies should focus on their structural modification, SAR and biogenic relationships.

## 1. Introduction

**Figure 1:**
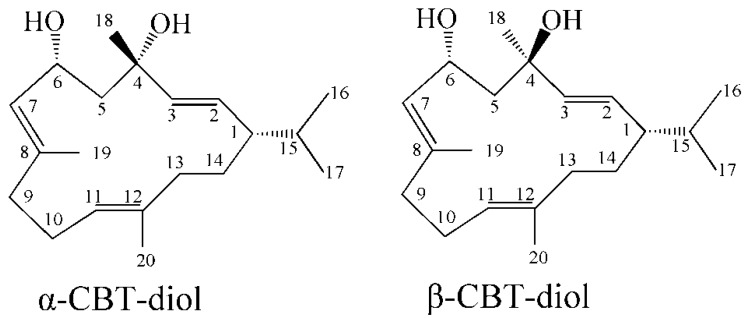
Chemical structures of cembratrien-diol (CBT-diol).

Tobacco leaves have a variety of chemical components, many of which are bioactive [1,2,3]. Cembranoids are macrocyclic diterpenes comprising four isoprene units bonded head-to-tail, and are natural products with a parent skeleton consisting of a 14-membered ring, three symmetrically distributed methyl groups and one isopropyl group; the cembranoid parent nucleus has a symmetry plane with an axis passing through C-1 and C-8 [4,5]. Cembranoid diterpenes are mainly present in the plants belonging to the genera *Nicotiana* and *Pinus*, and marine organisms (e.g., soft coral) [6], and they play a role in the continuous cropping obstacles of tobacco plants and the competitive survival of corals [4,7]. To the best of our knowledge, tobacco plants contain the highest content of cembranoid diterpenes. Since Roberts and Rowland [8] first identified the cembratrien-diol (CBT-diol, Figure 1) in tobacco, at least 89 cembranoid compounds [5,9] have been identified, including cembranoids, *nor*-cembranoids, *seco*-cembranoids, and cyclised cembranoids. Their functional groups include carbon–carbon double bonds and isopropyl, methyl, hydroxyl, hydroperoxyl, methoxy, epoxy and ketone groups. Cembranoid compounds are important aroma precursors in tobacco which are synthesised in tobacco gland hairs during its growth, and are mainly present in the surface exudates of leaves and flowers [5]. During the tobacco curing and ageing process, cembranoid compounds are degraded to produce solanone, solanofuran, norsolandione and other important flavour components [9,10,11]. Existing reviews on tobacco cembranoid compounds have mainly focused on their separation and identification, chemical structure, detection method, biosynthesis, chemical synthesis and biodegradation [10,11]. However, reviews on the recent research progress on the bioactivity of tobacco cembranoid diterpenes have not been published. Therefore, this present review summarises the bioactivity of tobacco cembranoid diterpenes and the effect of biocatalysis and semisynthesis on their bioactivity. This review will provide a reference for the extensive utilisation of tobacco cembranoid diterpenes.

## 2. Bioactivities of Tobacco Cembranoid Diterpenes

Although at least 89 tobacco cembranoid compounds have been reported, studies on the bioactivities of tobacco cembranoid diterpenes are mainly performed using CBT-diol [5].

### 2.1. Antimicrobial Activity

Tobacco cembranoid diterpenes have good antifungal [12,13,14,15,16,17], antibacterial [18], and antiviral [19,20,21] activities. The antifungal activity of tobacco cembranoid diterpenes was first reported in 1990 [12]. The CBT-diol could inhibit the spore germination of *Peronospora tabacina* [12], and the half-maximal inhibitory concentrations (IC_50_) of α-and β-CBT-diol on *P. tabacina* (adam) were 3.0 and 2.9 μg/cm^2^, respectively, indicating that the antimicrobial activity of β-CBT-diol was slightly stronger than that of α-CBT-diol [13]. Subsequently, CBT-diol was also found to inhibit the spore germination of *Colletotrichum lagenarium* with an IC_50_ of 6.3 μg/cm^2^ [14]. The antimicrobial activities of the 95% ethanol and *n*-hexane extracts of tobacco leaves on *Valsa mali* and 10 other plant pathogenic fungi were studied, and CBT-diol was speculated to be the main antimicrobial substance based on the antimicrobial properties of different material extracts and several pure substances [15]. Further studies showed that 80 mg/L cembranoid diterpenes completely inhibited the growth of *V. mali*, which might be related to the destruction of its endometrial structure [16]. Yan et al. [17] found that the IC_50_ of α-CBT-diol on *V. mali* was 18 mg/L, and its use against this fungus caused hyphal adhesion, uneven hyphal width, enlarged hyphal tip, thickened cell wall, retracted plasma membrane and disordered arrangement of intracellular mitochondria and other organelles, leading to the up- and downregulation of the expression levels of 94 and 107 genes, respectively. The gene ontology enrichment analysis of differentially expressed genes indicated that α-CBT-diol treatment significantly changed the expression of *V. mali* genes related to the redox process, tetrapyrrole binding, coenzyme binding, heme binding and iron binding [17].

In addition to the inhibitory effects on *Alternaria alternata*, *Aspergillus niger*, *Candida albicans*, *Fusarium chlamydosporum* and other fungi, CBT-diol also inhibited the growth of *Bacillus subtilis*, *Proteus vulgaris*, *Staphylococcus aureus* and other bacteria [18]. Studies also found that β-CBT-diol inhibited the replication of the human immunodeficiency virus (HIV) and could be used in the treatment of HIV-related neurocognitive disorders and HIV-induced inflammatory responses [19,20,21]. These reports indicate that tobacco cembranoid diterpenes have the potential to be developed as botanical fungicides [15,16,17] and anti-HIV drugs [19,20,21].

### 2.2. Insecticidal Activity

Although CBT-diol has good antimicrobial activity, cembratrien-ol (CBT-ol) has good insecticidal activity. The formation of CBT-diol by CBT-ol is catalysed by the cytochrome P450 (CYP) monooxygenase [5]. The CYP-suppressed transgenic tobacco plants showed a ≥19-fold increase in CBT-ol and a ≥41% decrease in CBT-diol, thus enhancing natural product-based aphid resistance [22]. Furthermore, overexpression of cembratrien-ol synthase gene using either trichome-specific CYP450 or Cauliflower mosaic virus 35S promoters greatly increased aphid resistance by promoting the accumulation of CBT-diols in tobacco plants [23]. Recent studies show that the recombinant *Escherichia coli* can be used to generate 78.9 ± 2.4 mg·L^−1^ CBT-ol in a 50 L bioreactor, and in vivo and in vitro bioactivity studies confirmed the insecticidal characteristics [24]. Therefore, tobacco cembranoid diterpenes can be researched for the development of insecticides due to their insecticidal activity [22,23,24].

### 2.3. Cytotoxic Activity

The cytotoxic activity of tobacco cembranoid diterpenes was first reported in 1985 [25]. Saito et al. [25] isolated and identified α-and β-CBT-diols from cigarette smoke and found that they inhibited the 12-*O*-tetradecanoylphorbol-13-acetate (TPA)-induced expression of Epstein–Barr (EB) virus early antigen in lymphoma cells with IC_50_ values of 25.2 and 21.9 μM, respectively, indicating that the cytotoxic activity of β-CBT-diol was stronger than that of α-CBT-diol. Further studies showed that the cytotoxic mechanism of CBT-diol might be related to its inhibitory effect on protein kinase C (PKC) and phospholipid metabolisms [26]. In contrast to most antineoplastic agents, β-CBT-diol did not show cytotoxicity even at a very high concentration (100 μM) [27]. Nacoulma et al. [28] isolated a mixture of cembranoid compounds from the leafy galls of tobacco infested by *Rhodococcus fascians*, and found that these compounds induced cell enlargement, slowed cell division, and altered the nuclear morphology and the polymerisation and stability of microtubules, and inhibited the proliferation of human glioma cells. At higher concentrations, these compounds also induced defects in cell mitosis, polyploidisation and apoptosis [28]. The molecular docking of tobacco cembranoid diterpenes with molecules such as cyclin-dependent protein kinase 2 and 6, PKC, vascular endothelial growth factor receptor 2, DNA topoisomerase II and tubulin led to speculation that its cytotoxic activity was related to molecular binding to its target receptor [29]. Recently, α-CBT-diol was identified as a novel angiogenesis inhibitory lead for the control of breast malignancies [30]. This study shows evidence and the potential of α-CBT-diol as a potent angiogenesis modulator, which targets the vascular endothelial growth factor receptor 2 (VEGFR2). In vitro, α-CBT-diol significantly reduced the activated VEGFR2 levels of the multiple breast cancer cell lines [30]. Additionally, α-CBT-diol semisynthetic analogues can be used as novel c-Met inhibitors for the control of the c-Met-dependent breast malignancies [31]. Recently, our study indicated that α-CBT-diol exhibited obvious inhibitory effects on the growth and colony-forming rate of liver hepatocellular (HepG2) cells and induced their apoptosis [32].

The cytotoxic activity of cembranoid diterpenes may be related to their inhibitory effect on PKC and phospholipid metabolism [26], regulation of the polymerisation and stability of microtubules [28], binding to target receptors [29] and potent angiogenesis modulation through targeting VEGFR2 [30] and c-Met inhibitors [31]. Additionally, the analysis of the structure–activity relationship (SAR) of tobacco cembranoid diterpenes showed that the C6 hydroxyl group, C11–C12 double bonds and other macrocyclic double bonds were critical to maintaining their cytotoxic activity [26]. Therefore, tobacco cembranoid diterpenes are a potential lead useful for future use in the development of cytotoxic drugs [33].

### 2.4. Neuroprotective Activity

Ferchmin and his collaborators conducted numerous studies that revealed the neuroprotective activity and mechanism of tobacco cembranoid diterpenes, which was first reported in 2001 [34]. Ferchmin et al. [34] demonstrated that tobacco cembranoid diterpenes inhibited the behavioural sensitivity of mice administered nicotine and blocked the functions of several nicotinic acetylcholine receptors (nAChR). Subsequently, it was also found that β-CBT-diol protected hippocampal slices against the *N*-methyl-D-aspartate (NMDA)-induced excitatory neurotoxicity as an antagonist of the nAChR [35]. Although both tobacco cembranoid diterpenes and nicotine exhibit neuroprotective activity, they accomplish it by different cellular signal transduction pathways. Specifically, the neuroprotective activity of cembranoid diterpenes is mediated by inositol trisphosphate 3-kinase (PI3K), L-type calcium channels and calmodulin-dependent protein, whereas that of nicotine is induced by PI3K, extracellular signal-regulated kinase 1/2 (ERK-1/2) and PKC [35]. Although β-CBT-diol cannot increase the total ERK-1/2 phosphorylation level, it increases the phosphorylation of Akt/PKB at ERK-1/2 activation sites and inhibits the β-phosphorylation of glycogen synthase kinase 3 at ERK-1/2 inhibitory sites [35]. The activation of the β-CBT-diol-mediated PI3K/Akt anti-apoptotic signalling pathway requires the activation of α_7_ nAChR and the direct inhibition of α_4_β_2_ nAChRs [35,36]. At the same time, β-CBT-diol also inhibits the nicotine-induced withdrawal behaviour of the planarian worm [37]. Therefore, the neuroprotective activity of tobacco cembranoid diterpenes is mainly mediated by nAChR-mediated anti-apoptosis and prevention of excitatory neuronal death [38].

Beta-CBT-diol protects rat hippocampal slices from organophosphate insecticide poisoning [39,40,41]. Parathion is an organophosphorus pesticide and paraoxon is its active metabolite. The use of β-CBT-diol to treat rat hippocampal slices could attenuate the damage to neuronal function, and the half-maximal effective concentration (EC_50_) of β-CBT-diol was 0.8 μM [39]. Beta-CBT-diol significantly reduced the damage to hippocampal slices induced by diisopropyl fluorophosphate, an analogue of the neurotoxic agent sarin, and 60 nM β-CBT-diol repaired 50% of the damage caused to the hippocampus slices [40]. It is noteworthy that α-CBT-diol did not exhibit neuroprotective activity against diisopropyl fluorophosphate [40]. The preliminary SAR and pharmacophore model analysis showed that the hydrophobic ring surface of tobacco cembranoid diterpenes bound with the hydrophobic patch of the receptors, and the electronegative atom (oxygen or sulphur) of the hydrophobic ring could bind to the electrically positive groups at the receptor binding site [40]. The use of β-CBT-diol 1 h prior to treatment with diisopropyl fluorophosphate or 24 h after treatment significantly reduced the neuronal death and inflammatory responses in the hippocampal CA1 region [41]. Beta-CBT-diol reduced the extent of the stroke-induced brain damage by inhibiting the expression of intercellular adhesion molecule-1 and restoring the phosphorylation of Akt [42]. A recent study has indicated that β-CBT-diol demonstrates a therapeutic effect in the rat 6-hydroxydopamine-induced Parkinson’s disease model in vivo and in 6-hydroxydopamine-challenged neuro-2a cells in vitro [43].

Tobacco cembranoid diterpenes have excellent neuroprotective activity and could be used to develop drugs for the treatment of nerve damage caused by organophosphorus pesticides [36,37,38,39], Alzheimer’s disease and Parkinson’s disease [43,44,45,46]. Recent studies have shown that β-CBT-diol penetrates the blood–brain barrier to reach the brain to play a neuroprotective role, and pharmacokinetic studies of tobacco cembranoid diterpenes would accelerate the development of neuroprotective drugs [47]. At the same time, because the tobacco cembranoid diterpenes inhibit nicotine behavioural sensitivity [34], they could also be investigated for further development of smoking cessation products.

## 3. Effects of Biocatalysis on Bioactivities of Tobacco Cembranoid Diterpenes

Biocatalysis of tobacco cembranoid diterpenes have been shown to yield cembranoid compounds with excellent cytotoxic and neuroprotective activities [5]. Biocatalysis refers to the process that uses enzymes or biological organisms (such as cells, organelles, and tissues) as a catalyst to initiate chemical conversions [48]. Compared with chemical catalysis, biocatalysis has the advantages of creating mild reaction conditions, strong specificity and high catalytic efficiency. The biocatalysis of tobacco cembranoid diterpenes, which includes hydroxylation, epoxidation and acetylation, mainly involves plant cells, microorganisms and enzymes. The biocatalysis of tobacco cembranoid diterpenes was first reported in 1987 [27]. As far as we know, cembranoid diterpenes are important aroma substances in tobacco [49]. To enhance the aroma of substances from fermented tobacco leaves, *Bacillus megaterium* NH5, obtained from the soil, was used to convert α-CBT-diol into two triols, and the addition of these two substances to cigarettes at a concentration of 0.0005% significantly enhanced the aroma of cigarettes and reduced the irritant effects of the smoke on the throat and lungs [50,51]. The conversion of CBT-diol was catalysed at different pH values using *Nicotiana sylvestris* and *Tripterygium wilfordii* cell suspension systems, and α-CBT-diol was converted into 10α-hydroxy-α-CBT-diol, 10β-hydroxy-α-CBT-diol, 12α-hydroxy-α-CBT-diol, (11S,12S)-epoxy-α-CBT-diol and 13α-hydroxy-α-CBT-diol [52,53]. *Bacillus* sp. NC5, *Bacillus* sp. NK7 and *Bacillus* sp. NK8 converted α-CBT-diol into various triol substances, and α-CBT-diol and these triols showed inhibitory activity on the human prostate cancer cell line PC-3M in the range of 10–50 nM [48]. β-CBT-diol could be converted to (11S, 12S)-epoxy-β-CBT-diol [52] using the *T. wilfordii* suspension cell system. *Mucor ramannianus* ATCC 9628 and *Cunninghamella elegans* ATCC 7929 converted β-CBT-diol to 10S and 11S-epoxy compounds [54]. Recently, *Novosphingobium* sp. HII-3, isolated from cured tobacco leaf, was confirmed to degrade the CBT-diol to farnesal [55]. β-CBT-diol was converted into triol compounds CYP450s [56,57], and the chemoenzymatic route to oxyfunctionalised cembranoids was facilitated by substrate and protein engineering [58].

## 4. Effects of Semisynthesis on Bioactivities of Tobacco Cembranoid Diterpenes

Semisynthesis of tobacco cembranoid diterpenes has been shown to yield cembranoid compounds with excellent cytotoxic activities [5]. Semisynthesis refers to a chemical synthesis method which uses natural animal-, plant- or microorganism-derived substances as the starting material for the synthesis of products, and desirable starting materials usually possess the basic skeleton, the majority of the functional groups or the desired configuration of the final product. The first total synthesis of CBT-diol was achieved in 1990 using diastereoselective [5,7] Witting ring contraction [59]. Subsequently, Wahlberg and Eklund [60] reported a variety of semisynthetic reactions of tobacco cembranoid diterpenes, including the oxidation of C7=C8 and C11=C12 double bonds, the allylic oxidation of C11=C12 double bonds, and the oxidation of C6 secondary alcohol to forms ketone and acid-catalysed rearrangement. Semisynthesis has demonstrated that C6 hydroxyl group, C11=C12 double bond and other macrocyclic double bonds of CBT-diol are the key structural elements of their cytotoxic activity. Among them, the acylation and oxidation of the C6 hydroxyl group to the corresponding ketone and alcohol or dehydration and rearrangement of C6 hydroxyl group reduces the cytotoxic activity of CBT-diol. The epoxidation of C11=C12 double bonds or the oxidation of C11=C12 double bonds into C11 or C12 hydroperoxides reduced the cytotoxic activity, and the saturation of CBT-diol macrocyclic double bond would lead to the loss of its cytotoxic activity [27].

El Sayed and his collaborators [48] synthesised the β-CBT-diol carbamate analogues and found that these compounds had anti-invasive activities against prostate PC-3M cancer cells at the concentrations of 10–50 nM. A series of compounds were synthesised via esterification, oxidation and halogenation of α-CBT-diol, and these compounds were shown to have excellent anti-proliferative activity against the highly malignant +SA mammary epithelial cells [54]. Alpha-CBT-diol was used to synthesise its carbamate analogues, and the compounds exhibited excellent cytotoxic activity against MDA-MB-231 breast cancer cells [31]. Thus, α-CBT-diol carbamate analogues can be used as novel c-Met inhibitors for the control of c-Met-dependent breast malignancies. Baraka et al. [61] isolated 4-methoxy-β-CBT-diol from fresh tobacco leaves and found that it exhibited excellent anti-migration activity on prostate cancer cells and its products of C=C8 double bond epoxidation showed excellent anti-migration activity on the highly metastatic prostate cancer cell lines PC-3 and PC-3M-CT^+^. Therefore, the semisynthetic reaction of tobacco cembranoid diterpenes have yielded some cembranoid compounds with excellent cytotoxic activity and its semisynthetic studies have shown that the C6 hydroxyl group, C11=C12 double bonds and other macrocyclic double bonds are the cytotoxic active groups of cembranoid diterpenes [27].

## 5. Conclusions and Future Perspectives

To date, studies on the excellent antimicrobial, insecticidal, cytotoxic and neuroprotective activities of tobacco cembranoid diterpenes have revealed that these compounds have the potential to be developed as botanical fungicides, cytotoxic drugs, as well as drugs for the treatment of HIV, Alzheimer’s disease, Parkinson’s disease and other neurodegenerative diseases. Additionally, biocatalysis and semisynthesis of tobacco cembranoid diterpenes have yielded cembranoid compounds with excellent cytotoxic and neuroprotective activities.

This review shows that tobacco cembranoid diterpenes have attracted more and more attention due to their unique chemical structure and good biological activity. The structural modification of tobacco cembranoid diterpenes is of great importance. However, there are relatively few studies on the SAR of tobacco cembranoid diterpenes. Therefore, future studies should focus on elucidating the SAR of tobacco cembranoid and developing cembranoid diterpene derivatives with high efficiency, low toxicity and stable structures. Further studies should also focus on the biogenic relationships of various skeletons and structural types of tobacco cembranoid diterpenes, deepening our understanding of the correlation between tobacco cembranoid diterpenes and laying a foundation for related studies of organic synthesis, pharmacochemistry and biosynthesis.
